# Developmental and Individual Differences in the Neural Processing of Dynamic Expressions of Pain and Anger

**DOI:** 10.1371/journal.pone.0093728

**Published:** 2014-04-04

**Authors:** Manuela Missana, Maren Grigutsch, Tobias Grossmann

**Affiliations:** 1 Early Social Development Group, Max Planck Institute for Human Cognitive and Brain Sciences, Leipzig, Germany; 2 Department of Neuropsychology, Max Planck Institute for Human Cognitive and Brain Sciences, Leipzig, Germany; University of Montreal, Canada

## Abstract

We examined the processing of facial expressions of pain and anger in 8-month-old infants and adults by measuring event-related brain potentials (ERPs) and frontal EEG alpha asymmetry. The ERP results revealed that while adults showed a late positive potential (LPP) to emotional expressions that was enhanced to pain expressions, reflecting increased evaluation and emotional arousal to pain expressions, infants showed a negative component (Nc) to emotional expressions that was enhanced to angry expressions, reflecting increased allocation of attention to angry faces. Moreover, infants and adults showed opposite patterns in their frontal asymmetry responses to pain and anger, suggesting developmental differences in the motivational processes engendered by these facial expressions. These findings are discussed in the light of associated individual differences in infant temperament and adult dispositional empathy.

## Introduction

Facial expressions play an important role in communicating emotions and in providing cues that guide behavior during social interactions [1]. Our ability to detect pain and anger in other people is likely to serve vital social and protective functions, enabling us to become aware of and respond appropriately to potentially harmful and dangerous situations. Observing someone expressing pain can convey harm and elicit empathic helping behavior, while observing someone expressing anger signals interpersonal threat and may result in readiness for aggression or a submissive flight response in the observer.

The facial expression that accompanies the experience of pain is highly specific and can be readily distinguished by observers from facial expressions of negative basic emotions such as anger and fear [2]. Expressing pain through facial expression is characterized by the lowering of the eyebrows and narrowing/closing of the eyes, raising of the cheeks, raising the upper lips, or vertically stretching the mouth open [2], [3].

The facial expression of anger is somewhat similar to the expression of pain as far as the eye regions are concerned, because it is also characterized by furrowed eyebrows and by staring eyes. However, in particular the mouth and cheek region differ across these two expressions, with angry expressions showing a closed mouth with tense lips [4].

The neural correlates of responding to pain in others have been studied extensively in adults [5]. This work provides evidence for shared representations in the human brain that are active both when adults feel pain and when they observe others in pain. On the basis of these findings, it has been argued that these shared representations constitute the neural basis of empathy for pain [6]. Adults' brain responses to pain in others have been examined in various experimental contexts such as (a) by knowing that another person was receiving a painful stimulation to the hand as indexed by a symbolic (arrow) cue [7], (b) by viewing body parts of actors in painful situations [8]–[11], and (c) by observing facial expressions of pain [12]–[15]. Across these different contexts painful situations systematically resulted in activation of the anterior cingulate cortex (ACC) and the anterior insula (AI) [5]. The notion that brain activation in these regions is a neural correlate of empathy for pain receives support from findings showing that brain responses to pain in others vary as a function of individual differences in empathic abilities, with individuals that score higher in empathy showing greater activation in their brain responses to pain [7], [14]. Furthermore, brain responses within these regions show a great level of specificity and indicate that adults discriminate between pain and other negative emotional states [16].

Adults' ability to discriminate between pain and other negative expressions has also been shown in recent event-related brain potential (ERP) studies [17], [18]. The ERP method provides precise information concerning the timing of brain processes associated with emotion perception. In prior work a general distinction has been made between *early* processes related to emotional attention as reflected in an early posterior negativity (EPN) and *late* evaluative processes reflected in a late positive potential (LPP) [19]. More specifically, in previous ERP studies it was found that whereas seeing angry facial expressions resulted in an enhanced EPN indexing increased perceptual (visual) processing related to the rapid detection of threatening faces [20], [21], facial expressions of pain elicited an LPP response that was enhanced in its amplitude compared to angry and fearful facial expressions [17], [18]. An enhanced LPP is thought to reflect increased evaluation of an emotionally arousing stimulus [19]. These studies thus provide evidence that the adult brain not only distinguishes between negative facial expressions but also shows an increased sensitivity and arousal to facial expressions of pain as indexed by an enhanced LPP. Similar ERP effects were observed when adults watched others in painful situations [22], supporting the notion that the neural processes reflected in this ERP component can be flexibly triggered by observing others in pain even in the absence of overt facial cues.

From a functional perspective it is important to add that, apart from eliciting empathic responses, painful expressions may also serve an adaptive alarm function leading to the facilitation of defensive responses in the observer [23]–[25]. In line with this view, Yamada and Decety (2009) showed that pain detection was enhanced after subliminal priming with negative affective stimuli when compared to priming with positive affective stimuli. It has been argued that the perception of pain might therefore be associated with an activation of threat-related brain systems [25]. The notion that experiencing pain or observing pain in others evokes activity in threat-related brain systems has also been shown in fMRI studies that found increased activation of the amygdala in response to pain [12], [15].

From a developmental perspective, it has been argued that it may be adaptive for humans to respond sensitively to emotional expressions from early on in development [26]–[28]. There is evidence from behavioral and neural studies showing that infants from around 7 months of age can reliably discriminate between a variety of affective facial expressions [29]–[32]. For example, findings from studies using ERPs demonstrate that infants at the age of 7 months discriminate happy from fearful and angry expressions [33], [34], as indexed by differences in a negative component (Nc) elicited over anterior brain regions between 300 and 600 ms. Critically, evidence for 7-month-old infants' ability to discriminate between different negative emotional expressions has been provided by Kobiella and colleagues (2008). In this study, infants were presented with static angry and fearful facial expressions. Angry compared to fearful facial expressions elicited a larger fronto-central negativity in the time range from 300 to 600 ms [35]. An enhanced negativity over anterior brain regions in this time window is thought to reflect greater orientation and attention allocation to the stimulus [36], suggesting increased allocation of attentional resources to angry faces. Even though there is evidence that infants at the age of 7 months can discriminate between various facial expressions as reflected in amplitude modulations of the Nc, it is not well understood how the infant Nc component relates to the ERP components generally reported during emotion processing in adults (EPN and LPP) (see above). For example, there is evidence showing that although at the age of 7 months infants exhibit an enhanced Nc over anterior electrodes in response to angry faces, it is not until 12 months of age that infants, like adults, show an enhanced EPN over posterior (occipital) electrodes in response to angry faces [33]. Moreover, very little is known about how infants respond to facial expressions of pain and its neural correlates. Closing this gap in our understanding of emotional responding during infancy by studying infants' brain responses to expressions of pain is particularly pertinent given the role that responding to pain in others has played in the investigation of empathy in general [6], [9], [37], [38] and its development in particular [39]–[41].

In light of the work discussed above, three main questions were addressed in this study: (a) How does the neural processing of dynamic facial expressions of pain and anger compare between infants and adults; (b) Can infants discriminate between facial expressions of pain and anger; (c) Whether and how individual differences in dispositional empathy (adults) and temperament (infants) impact brain responses to these emotional expressions, and if so, what can this tell us about the function of those specific brain processes under investigation?

In order to examine these questions, we measured ERPs in response to dynamic facial expressions of pain and anger in adults and 8-month-old infants. In the present study, dynamic facial expressions were used because: (a) prior work with adults suggests an improved performance across a range of face perception tasks including face identity recognition and facial emotion recognition [42], [43], (b) dynamic facial expressions are thought to be more ecologically valid since this is how they are typically experienced during social interactions, and (c) infants may better attend to the dynamic presentations than watching static displays [44]–[46].

In addition, we assessed frontal EEG alpha power asymmetry to elucidate the motivational processes related to approach and withdrawal tendencies evoked by viewing these facial expressions. Approach and withdrawal are assumed to reflect basic motivational dimensions in human behavior [47]. Specifically, while approach motivation is linked to increased exploration of the social and physical environment, withdrawal motivation is associated with inhibition of exploration and most frequently related to negative affect [48]. With respect to the neural correlates of these motivational tendencies, Davidson [49], [50] proposed a model that links frontal EEG asymmetry to motivational tendencies and affective styles. Asymmetrical frontal brain activity in the alpha frequency band in adults and infants suggests that the lateralization of cortical activity measured at frontal electrode sites is associated with different motivational tendencies and can be seen as an index of approach or withdrawal motivations [49]–[54]. These studies show that approach motivation is associated with relatively greater left frontal cortical activation whereas relatively greater right frontal cortical activation is associated with withdrawal motivation. Specifically, frontal EEG alpha asymmetry research suggests that anger, while being a negatively valenced emotion, is typically associated with approach motivation, eliciting greater relative left frontal activation during anger-evoking events [55], [56].

Finally, in order to investigate individual (trait) differences in emotional sensitivity and its relation to the neural processing of pain and anger, we obtained information about adult dispositional empathy and infant temperament using questionnaires. This approach was informed by previous studies (a) with adults: demonstrating that individuals that score higher in empathy, as measured by an empathy questionnaire, exhibit greater activation in their brain responses to pain [7], [14], and (b) with infants: demonstrating that differences in emotion regulation abilities, as measured by a parental questionnaire (IBQ-R), were associated with differences in the brain responses to negative facial expressions [57]. The temperament of the infants, in particular approach- and withdrawal-related traits as measured by a parental questionnaire (IBQ-R), was found to be related to general differences in frontal EEG alpha asymmetry patterns in 7- to 9-month-old infants [58]. On the basis of these findings, we expected that infants' ERP and frontal EEG asymmetry responses to negative facial expressions would be similarly related to measures of infant temperament.

## Materials and Methods

### Adults

#### Participants

Twenty right-handed young adults aged between 21 and 28 years (10 female, *Median* age  = 25.5 years, *Range*  = 7 years) participated in the study. The participants had no prior history of psychiatric illness. Ethical approval was obtained from the Ethics Committee of the University of Leipzig. The participants provided written informed consent and were paid for their participation.

#### Stimuli

**Figure 1:**
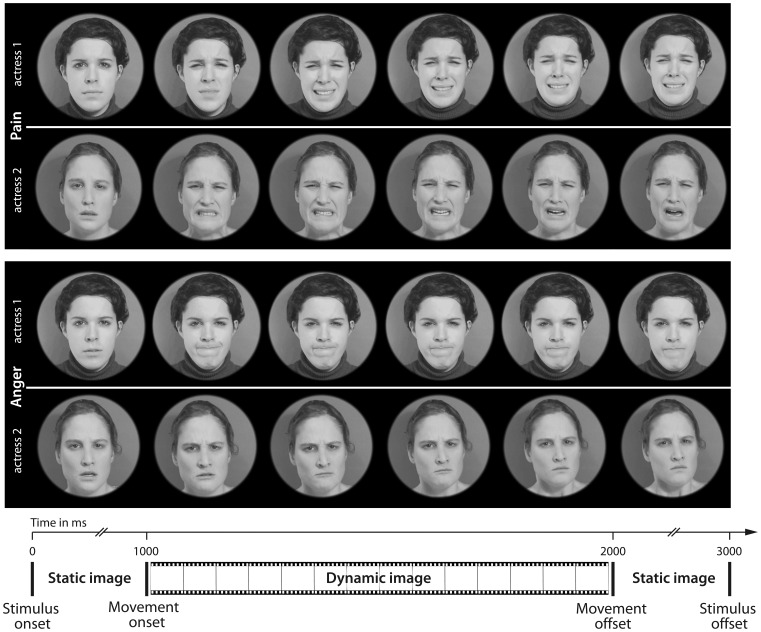
Examples of the stimuli. This figure shows representative examples of the stimuli. Single video frames of facial expressions of pain (top two rows) and anger (bottom two rows) for both actresses are shown.

The stimulus material consisted of video clips of dynamic facial expressions of pain and anger as well as happy and neutral expressions displayed by two actresses. In keeping with the stimulus presentation protocols of prior infant facial emotion processing ERP studies [34] each participant was presented with expressions of only one of the actresses. Happy and neutral facial expressions were presented but not used for analysis in order to avoid overwhelming the infants with negative expressions, thereby improving the testing atmosphere and reducing the dropout rate (ERP responses and frontal asymmetry responses to all four emotional expressions in infant and adults are provided in the supplementary information; see also Figures S1 and S2). Presenting negative expressions against a background of neutral and happy expressions also likely presents the infants with a more ecologically valid task, as in typical development they are thought to only infrequently encounter negative affect in daily interactions [59]. All stimuli were taken from a previously published study by Simon and colleagues [15], [60] and slightly modified (see Figure 1). The actresses provided written informed consent, transferring the copyright of the produced material to the research group [60]. The original video clips had a duration of 1 s and were cut backward from the peak of expression in order to control for different lengths, variability of exposure to the visual stimuli, and to assure that the peak of expression was captured within the clip [15], [60]. In addition, we analyzed the motion onset and overall amount of motion across emotional expressions based on a procedure by Pichon and colleagues [61]. Critically, this analysis showed that there were no systematic differences in the onset and overall amount of motion between the facial expression videos. In order to focus the participants' attention on the facial expressions, the original clips were edited by cropping external features such as the shoulders. All video clips had a duration of 3 s. Each clip consisted of a 1-s static image displaying a neutral expression followed by a 1-s dynamic expression followed by another 1-s static image displaying the peak of expression. The first static image was taken from the first frame and the second static image was taken from the last frame of the dynamic expression.

#### Procedure

The participants sat in a dimly lit, sound-attenuated, and electrically-shielded room facing a computer screen. They were instructed to attentively view the stimuli but no task was given in order to ensure that the data could be compared between adults and infants. The stimuli were presented in the center of the screen on a black background, using a 70-Hz, 17-inch computer screen at a distance of 70 cm. Each participant was randomly assigned to one of the two actresses. The presentation of either actress was counterbalanced. Before each video clip started, an alert signal sounded. Then a fixation cross appeared (for 1000 ms) on the screen to draw participants' attention to the center of the screen. This was followed by a black screen (for 300 ms) and then by the stimuli (3000 ms). The stimuli were presented in a pseudo-randomized order. The randomization was such that no expression was repeated more than once in a row in the course of the experiment. Participants viewed 41 trials of each facial expression. After the session, the stimuli were shown again and participants were asked to rate the facial expressions for arousal using the Self-Assessment Manikin (SAM) self-report scale [62].

#### Questionnaire

To assess individual differences in empathic abilities, participants filled out the self-report questionnaire Interpersonal Reactivity Index (IRI) [63]–[65]. The questionnaire consists of four sub-scales that are related to empathy. The two subscales empathic concern and personal distress are related to the emotional component of empathy. The perspective-taking subscale is related to the cognitive dimension of empathy, and the fantasy-empathy subscale represents the ability to identify with fictional characters in movies and novels [63]. Based on prior work with adults [7], we focused our analysis on only two subscales of the IRI, namely the empathic concern and the perspective-taking scales.

#### EEG measurement and ERP analysis

The EEG was recorded from 63 Ag/AgCl electrodes attached to an elastic cap (*EasyCap GmbH*, *Germany*) using the 10–20 system of electrode placement. The data were online referenced to the left mastoid and offline re-referenced to the algebraic mean of the left and right mastoid electrode. The horizontal electrooculogram (EOG) was bipolarly recorded from two single electrodes placed at the outer canthi of both eyes and the vertical EOG from electrodes on the infra- and supraorbital ridges of the right eye. The EEG was amplified using a 72-channel REFA8 amplifier (Twente Medical Systems International) in the frequency band between DC and 67.5 Hz and digitized at a rate of 250 Hz. Electrode impedances were kept below 5 kΩ. Data processing for ERP analysis was performed using an in-house software package EEP, commercially available under the name EEProbe™ (Advanced Neuro Technology, Enschede). The raw EEG data were bandpass filtered between 0.3 and 20 Hz, and the recordings were segmented into epochs time-locked to the stimulus onset, lasting from 200 ms before onset until the offset of the video clips (total duration 3200 ms). The epochs were baseline corrected by subtracting the average voltage in the 200-ms baseline period (prior to video onset) from each post-stimulus data point. Data epochs were rejected off-line whenever the standard deviation within a gliding window of 200 ms exceeded 60 μV in any of the two bipolar EOG channels and 50 μV at EEG electrodes (F3, Fz, F4, C3, Cz, C4, T7, T8, P3, Pz, P4, O1, O2). At each electrode, artifact-free epochs were averaged separately for angry and painful facial expressions to compute the ERPs. The average number of epochs included in the final analyses was 37.1 for angry facial expressions and 37 for painful expressions. Statistical analyses were based on the visual inspection of the ERP waveforms and prior work focusing on the EPN [17], [20] and LPP [17]. On the basis of this information, mean amplitude effects were assessed over a posterior occipital region (O1, O2) during an early time window from 250 to 350 ms post movement onset (EPN) and at an anterior ROI comprising frontal and central electrodes (F3, FZ, F4, C3, CZ, C4) during a later time window from 400 to 500 ms post movement onset (LPP). Mean amplitude effects were compared between facial expressions using paired-sample t-tests.

#### EEG measures of asymmetrical activation

Frequency analysis of the EEG data was performed using the *FieldTrip* software [66] in combination with custom-made MATLAB scripts. The raw EEG data were highpass filtered with a cut-off frequency of 1 Hz in order to reduce slow drifts and remove DC components. The recordings were segmented into epochs of 4000 ms duration, lasting from 1000 ms prior to stimulus onset until video offset. Epochs were visually inspected and excluded from further analyses if they were contaminated by large non-stereotyped artifacts (e.g., gross muscle activity or movement artifacts). Remaining stereotyped artifacts (originating e.g., from eye blinks or eye movements, tonic muscle activity, or pulse artifacts) were corrected using a signal processing procedure [67] based on Independent Component Analysis (ICA). The segmented EEG data were decomposed into 60 independent components (ICs) by application of the symmetric FastICA algorithm. ICs representing physiological or electrode artifacts were identified by visual inspection of the components' scalp topographies, frequency spectra, and single-trial time courses. They were removed from the data before back projection to the electrode space. For the analysis of event-related oscillations, time-frequency representations of artifact-cleaned single trials were computed using Morlet wavelets with a width of 5 cycles. Mean alpha power during the processing of facial expressions was estimated by averaging the squared magnitude of the complex wavelet transform coefficients across trials (separately for angry and painful facial expression), over time points during the presentation of the dynamic stimuli (0–1000 ms post movement onset) and frequency bins (8–13 Hz). Mean alpha power values were then log-transformed using the natural logarithm function (ln) to normalize their distribution. EEG alpha power asymmetry scores were calculated for the mid-frontal (F3, F4) and lateral frontal (F7, F8) regions. The scores were obtained by subtracting left log-transformed alpha power values from the corresponding right log-transformed values (ln(right) – ln(left)). It has been shown that increases in alpha power are associated with decreased cerebral activation and vice versa [68], [69]. The asymmetry score reflects the power in one hemisphere relative to the power in the opposite hemisphere. Higher scores on this metric suggest relatively greater left activity [70]. For comparison reasons, as in prior studies [53], asymmetry scores were also computed for the central region (C3, C4) and the parietal region (P3, P4).

### Infants

#### Participants

The final sample consisted of 20 8-month-old infants aged between 247 and 271 days (10 females, *Median* age  = 259 days, *Range*  = 24 days) and all came from a middle-class background in a medium-sized German city. The infants were born full term (between 37 and 41 weeks) and had a normal birth weight (>2500 g). Twenty additional infants were tested but had to be excluded from the final sample due to fussiness (n = 6) or too many artifacts (n = 14). Ethical approval was obtained from the Ethics Committee of the University of Leipzig. All parents provided written informed consent prior to the study and were paid for their children's participation.

#### Stimuli

The stimuli were the same as those used in the adult experiment (see above).

#### Procedure

The infants sat on their parent's lap during testing. Parents were asked not to talk to or interact with their infant during the course of the experiment. Each participant was randomly assigned to one of the two actresses. The presentation of either actress was counterbalanced.

In order to attract infants' attention to the screen, each facial expression video was preceded by a sound and a fixation cross (1000 ms). This was followed by a black screen (300 ms) and then the stimuli (3000 ms). During the inter-stimulus interval infants were presented with an abstract screensaver for the purpose of keeping infants' attention. The inter-stimulus interval lasted at least 1000 ms and varied depending on infants' attentiveness, as stimulus presentation was controlled by an experimenter in such a way that stimuli were only presented when infants were looking at the screen. In order to control for infants' attention to the stimuli, infants' were video monitored throughout the EEG recording. The EEG session ended when the infant became restless or inattentive. The mean number of trials seen per condition was 15.08. The criterion for the minimum number of trials for inclusion in the final ERP average was 5 artifact-free trials per condition. The mean number of trials included in the ERP average per condition was 10.78. While the minimum number of trials and the mean number of trials to be included in the final analysis might appear lower than in previous studies, please note that we used dynamic video stimuli in the current design that were substantially longer (about 2 seconds longer) than those used in prior research with static facial displays of emotion and applied a strict criterion for inclusion, which required the entire trial epoch (3200 ms) to be artifact free [71]. This and the additional use of neutral and happy dynamic stimuli likely accounts for the smaller trial numbers in the current study.

#### Questionnaire

Parents were asked to fill out a temperament questionnaire (Infant Behavior Questionnaire in its revised form, IBQ-R, German version). The IBQ-R is the most commonly used questionnaire to assess differences in temperament in infants. The IBQ-R consists of 14 subscales that cover a wide range of temperamental traits: approach, vocal reactivity, high intensity pleasure, smiling and laughter, activity level, perceptual sensitivity, sadness, distress to limitations, fear, falling reactivity/rate of recovery from distress, low intensity pleasure, cuddliness, duration of orienting, soothability [72]. In accordance with previous studies that investigated the influence of temperament on infants perception of emotions and frontal EEG alpha asymmetry [57], [58], temperament analyses in the present study were limited to two dimensions of infant temperament, namely, ‘negative emotionality’ (as indexed by the subscales fear, sadness, distress to limitations, recovery from distress) and approach oriented temperament (as indexed by the subscales approach and duration of orienting).

#### EEG measurement and ERP analysis

The EEG was recorded from 27 Ag/AgCl electrodes attached to an elastic cap (*EasyCap GmbH, Germany*) using the 10–20 system of electrode placement. The data were online referenced to the CZ electrode and offline re-referenced to the algebraic mean of the left and right mastoid electrode. The horizontal electrooculogram (EOG) was recorded from two electrodes (F9, F10) which are part of the cap located at the outer canthi of both eyes. The vertical EOG was recorded from an electrode on the supraorbital ridge (Fp2) which is part of the cap and an additional single electrode on the infraorbital ridge of the right eye. The EEG was amplified using a Porti-32/M-REFA amplifier (Twente Medical Systems International) and digitized at a rate of 500 Hz. Electrode impedances were kept between 5 to 20 kΩ.

Further processing was done analogously to the adult data analyses with the exception that infant data epochs were rejected off-line whenever the standard deviation within a gliding window of 200 ms exceeded 100 μV in any of the two bipolar EOG channels and 80 μV at EEG electrodes (F3, Fz, F4, C3, Cz, C4, T7, T8, P3, Pz, P4, O1, O2). At each electrode, artifact-free epochs were averaged separately for angry and painful facial expressions to compute the ERPs.

Statistical analyses were based on the visual inspection of the ERP waveforms and prior work focusing on the Nc [35]. On the basis of this information, mean amplitude effects were assessed at an anterior ROI comprising frontal and central electrodes (F3, FZ, F4, C3, CZ, C4) during a 500 to 600 ms time window post movement onset (Nc). Visual inspection of the infant ERP data revealed no clearly defined ERP components at occipital electrodes and no discernable amplitude differences between facial expressions at these electrodes. Mean amplitude effects were compared between facial expressions using paired-sample *t-*tests.

#### EEG measures of asymmetrical activation

Analyses for measuring infant EEG alpha asymmetry was done, with some exceptions, analogously to adult data analyses. Because infant data contained 50-Hz notch noises, a 50-Hz notch filter was applied after segmentation of the data. For artifact correction using the ICA procedure [67] the segmented EEG data were decomposed into 24 independent components. Power values were obtained in the alpha frequency band from 6 to 9 Hz. The alpha frequency band is lower in infants than in adults, therefor, as suggested in previous work, we studied alpha power ranging from 6 to 9 Hz [73]. The calculation of the EEG alpha power asymmetry scores was done analogously to the adult data.

## Results

### ERP Analysis

#### Adults

**Figure 2:**
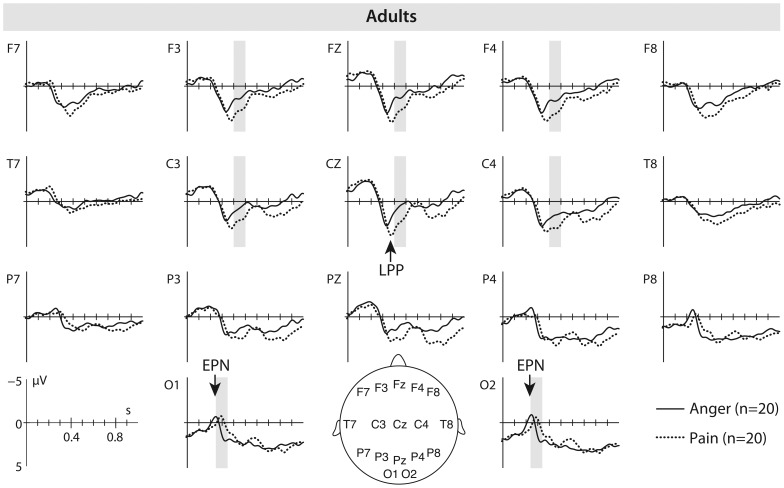
Adult ERP results. This figure shows the average event-related brain potentials (ERPs) time-locked to the movement onset in adults elicited by facial expressions of anger (solid line) and pain (dotted line) expressions. The time windows during which significant differences between the anger and pain condition were observed are marked in grey.

Our ERP analysis revealed a significant difference for the EPN in response to pain and anger facial expressions between 250 and 350 ms post movement onset at occipital electrodes, *t*(19) = 3.62, *p* = .002. Visual inspection of the ERP data indicated a difference in peak latency for the EPN in response to anger and pain expressions (see Figure 2). An additional analysis of peak latency effects during a 500 to 600 ms time window post movement onset revealed a significant difference in peak latency, *t*(19) = −2.23, *p* = .037, with the EPN to angry faces peaking earlier than the EPN to pain faces. Our ERP analysis further revealed a significant difference for the LPP in response to painful and angry facial expressions at fronto-central electrode sites between 400 and 500 ms after movement onset, *t*(19) = −2.97, *p* = .008. Specifically, the LPP elicited by facial expressions of pain was greater (more positive) in its amplitude (*M* = 2.62 μV; *SE* = 0.61) than the LPP elicited by facial expressions of anger (*M* = 1.13 μV; *SE* = 0.60) (see Figure 2). Behavioral arousal ratings obtained after the EEG measurement showed that there was a significant difference in arousal between pain and anger, with pain expressions (*M* = 3.45; *SE* = 0.153) rated as being more arousing than anger expressions (*M* = 2.85; *SE* = 0.182), *t*(19) = −2.69, *p* = .014. No significant correlations between the arousal ratings for facial expressions of pain and LPP responses to facial expressions of pain were found.

#### Empathy self-report questionnaire

**Figure 3:**
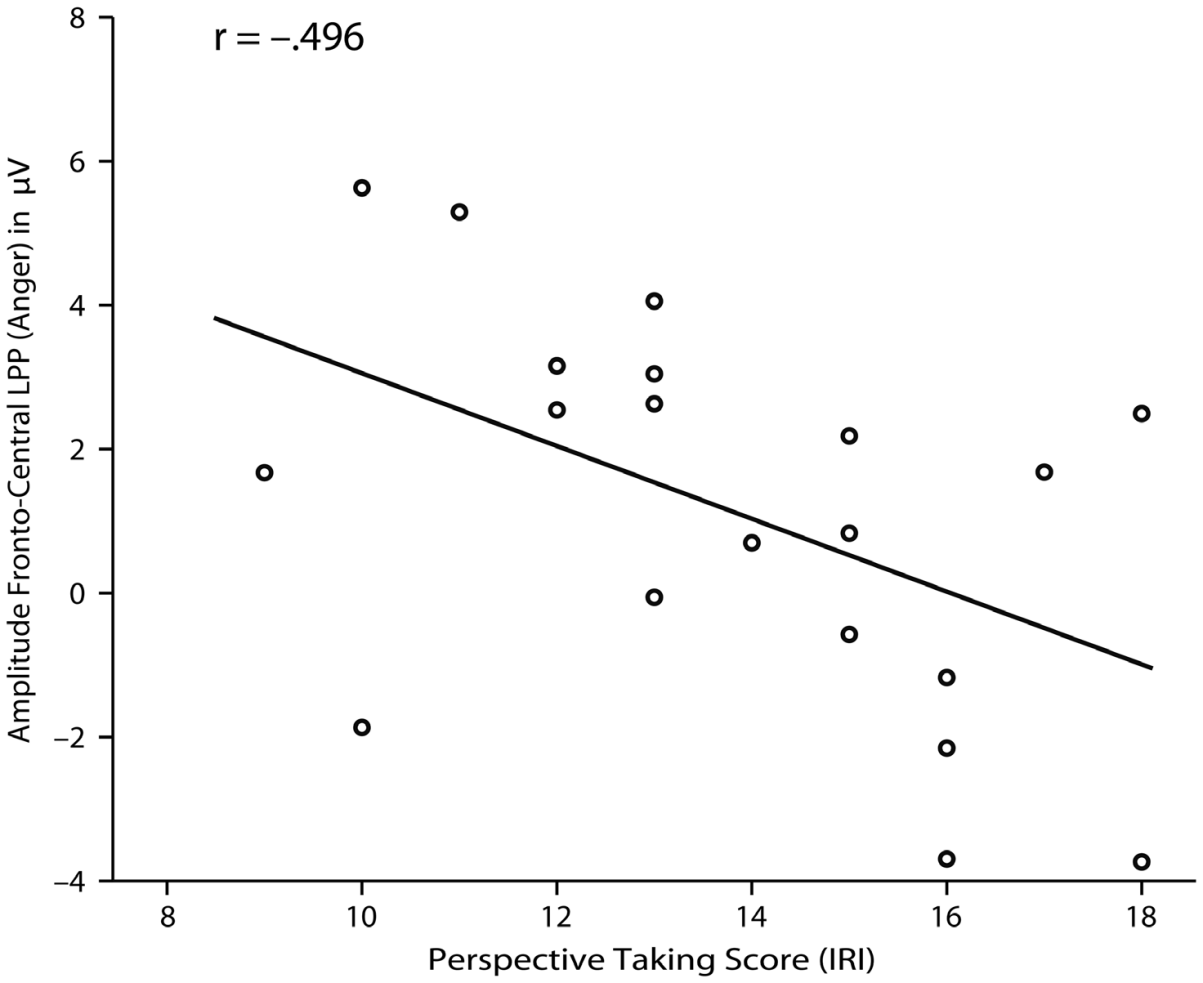
Correlation for adult ERP results. This figure shows the correlation between the amplitude of adults' brain responses to facial expressions of anger at fronto-central electrodes and individual perspective-taking scores as measured by the IRI (the correlation was significant on the *p*<0.05 level).

Our analysis revealed a significant negative correlation between the ERP response to facial expressions of anger at fronto-central electrodes and the perspective-taking score as measured by the IRI (*r* = −.496, *p* = .026) (see Figure 3). The perspective-taking score is an index of a person's ability and motivation to adopt another person's point of view [63]. Specifically, the correlation was such that the higher adults rated themselves as possessing the ability and motivation to take another person's perspective, the smaller the amplitude of the LPP to angry expressions. There were no correlations between the ERPs in response to pain facial expressions and the IRI.

#### Infants

**Figure 4:**
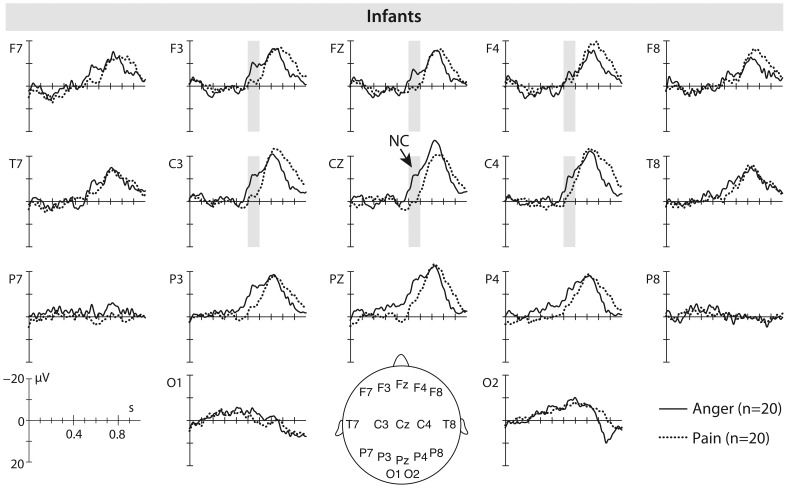
Infant ERP results. This figure shows the average event-related brain potentials (ERPs) time-locked to the movement onset in 8-month-old infants elicited by facial expressions of anger (solid line) and pain (dotted line). The time windows during which significant differences between the anger and pain condition were observed are marked in grey.

Our ERP analysis revealed that, unlike adults, watching dynamic facial expressions of pain and anger resulted in a negativity elicited over anterior brain regions in infants that differed in its amplitude between facial expressions (see Figure 4). Specifically, infants discriminated between the two negative expressions as revealed by a significant difference between the ERP response to facial expressions of pain and anger between 500 and 600 ms after movement onset, *t*(19) = −2.64, *p* = .016. The ERP response to facial expressions of anger was more negative in its mean amplitude (*M* = −8.19 μV; *SE* = 2.42) than the ERP response to facial expressions of pain (*M* = −1.89 μV; *SE* = 1.79). No ERP differences between expressions were found at occipital electrodes.

#### Infant temperament questionnaire

**Figure 5:**
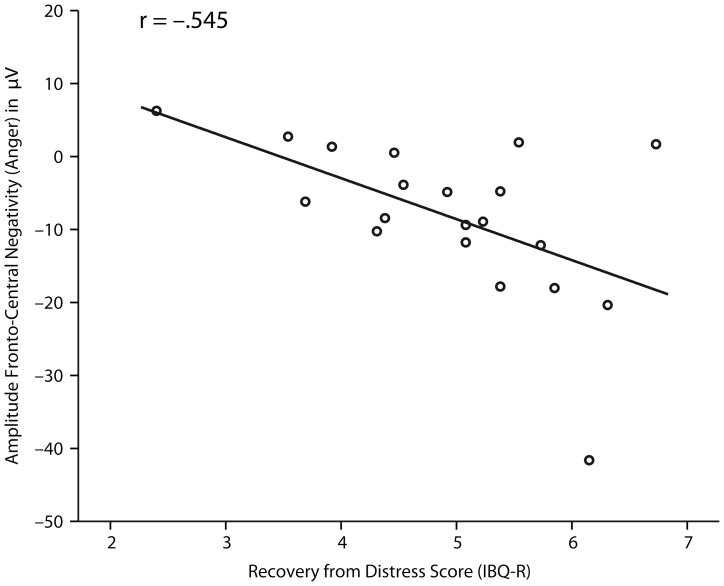
Correlation for infant ERP results. This figure shows the correlation between the amplitude of infants' brain responses to facial expressions of anger at fronto-central electrodes and individual recovery from distress scores as measured by the IBQ-R (the correlation was significant on the *p*<0.05 level).

Our analysis revealed a significant negative correlation between the ERP response to facial expressions of anger and the recovery from distress score as measured by the IBQ-R, *r* = −.545, *p* = .013 (see Figure 5). The recovery from distress score reflects parents' ratings of their infants' ability to regulate emotions and regain calm after distress [72]. Specifically, the correlation was such that the higher the parents rated their infants' ability to regulate and recover from distress, the more negative the amplitude of the ERP in response to angry expressions. Other subscales of the IBQ-R were not related to infants' ERP responses to anger and pain facial expressions.

### Frontal EEG alpha asymmetry analysis

#### Adults

Our analysis revealed a significant difference between the frontal EEG alpha asymmetry scores in response to facial expressions of anger and pain at lateral-frontal electrodes (F7, F8), *t*(19) = 3.10, *p* = .006. Facial expressions of anger were found to result in greater (positive) frontal EEG alpha asymmetry scores indicative of a greater relative left frontal activation, while facial expressions of pain were found to result in smaller (negative) frontal EEG alpha asymmetry scores indicative of greater relative right frontal activation (see Table 1). A similar effect with greater relative left frontal activation in response to anger as compared to greater relative right frontal activation to pain was also observed at mid-frontal electrodes (F3, F4) where the difference between the frontal EEG alpha asymmetry scores in response to facial expressions of anger and pain was marginally significant, *t*(19) = 1.90, *p* = .072 (see Table 1). For central (C3, C4) and parietal regions (P3, P4) there were no differences in EEG alpha asymmetry scores between expressions.

**Table 1:** Frontal EEG alpha asymmetry scores.

			Condition			
	*N*	Electrodes	Anger	Pain	*t*	*p*
**Adults**	20	F7/F8	0.013±0.42	−0.046±0.41	3.102	0.006**
		F3/F4	0.074±0.17	0.047±0.20	1.908	0.072+
**Infants**	20	F3/F4	−0.009±0.13	0.054±0.20	−2.111	0.048*

This table shows the mean (± standard deviation) of the frontal EEG alpha asymmetry scores during the presentation of facial expressions of anger and pain. Please note that higher numbers indicate greater relative left-side activation.

**p<.01.

*p<.05,+*p* = marginal significant.

#### Empathy self-report questionnaire

**Figure 6:**
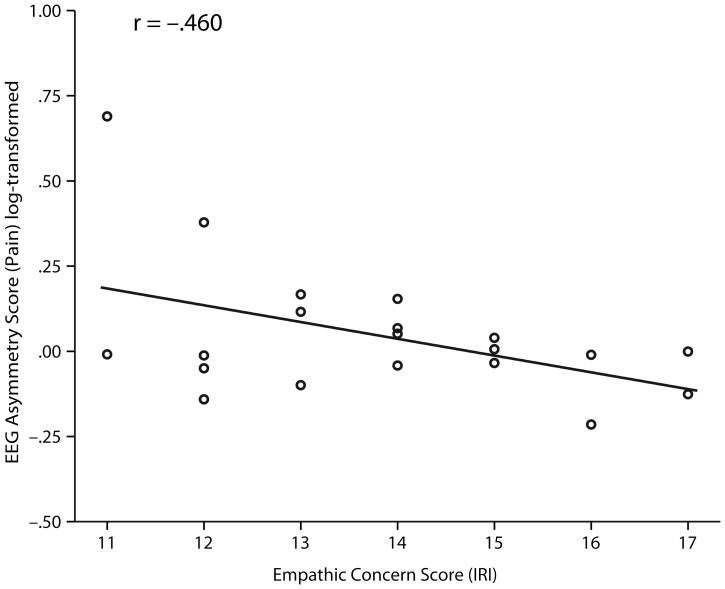
Correlation for adult frontal EEG alpha asymmetry results. This figure shows the correlation between frontal EEG alpha asymmetry observed in adults during the presentation of facial expressions of pain and individual empathic concern scores as measured by the IRI (the correlation was significant on the *p*<0.05 level).

Our results revealed a significant negative correlation between individuals' frontal EEG alpha asymmetry scores (F3, F4) in response to facial expressions of pain and empathic concern scores as measured by the IRI, *r* = −.460, *p* = .041 (see Figure 6). The empathic concern score refers to the individual's degree of participating in other people's emotions, and experiencing feelings of sympathy and concern for others [63], [64]. Specifically, the observed correlation was such that the higher adults rated themselves as possessing the ability and motivation to experience feelings of sympathy and concern for others, the more right lateralized their frontal EEG alpha asymmetry score, indexing a greater motivational tendency to withdraw.

#### Infants

Our results revealed a significant difference between the frontal EEG alpha asymmetry scores in response to facial expressions of anger and pain at mid-frontal electrodes (F3, F4), *t*(19) = −2.11 *p* = .048 (see Table 1). Contrary to the results in adults reported above, in infants, viewing facial expressions of pain was associated with greater (positive) frontal EEG alpha asymmetry scores, while viewing facial expressions of anger was associated with smaller (negative) frontal EEG alpha asymmetry scores. This pattern reflects greater relative left frontal activation when processing pain, indexing the motivational tendency to approach and greater relative right hemisphere activation when processing anger, indexing the motivational tendency to withdraw. There were no differences between the EEG alpha asymmetry scores at the lateral-frontal electrodes (F7, F8), *t*(19) = .2 *p* = .841. Furthermore, EEG alpha asymmetry at central (C3, C4) and parietal (P3, P4) electrodes did not differ between expressions.

#### Infant temperament questionnaire

There were no correlations between frontal EEG alpha asymmetry scores in response to anger and pain and infant temperament scores as measured by the IBQ-R.

## Discussion

**Figure 7:**
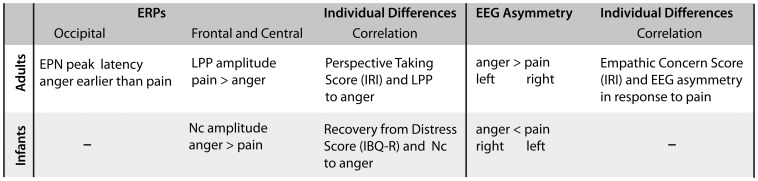
Overview of findings. This table provides an overview of the findings of the current study.

The current study investigated the neurodevelopment of processing dynamic expressions of pain and anger. To our knowledge, this is the first study to compare infants' and adults' emotion processing by measuring both ERPs and frontal EEG alpha asymmetry in brain activation and linking differences in brain response to individual differences in infant temperament and adult dispositional empathy. Our study demonstrates that taking such a multi-measure (ERP, frontal EEG alpha asymmetry, temperament, empathy) and multi-method (EEG/ERPs and questionnaire methods) approach is very useful in investigating developmental differences between infants and adults and may contribute to a more comprehensive understanding of the development of emotion processing (see Figure 7 for an overview of the ERP and frontal EEG alpha asymmetry findings in the current study).

The ERP results indicate that the brain processes elicited by expressions of pain and anger differ substantially between infants and adults. Our ERP data show that, in adults, facial expressions elicited an EPN at posterior (occipital) electrodes that peaked earlier in response to angry faces than to pain faces, suggesting that the perceptual (visual) processing was facilitated in response to angry faces. This facilitation effect evident in the response to angry faces is in line with prior work with adults showing that angry faces as evolutionary important signals of interpersonal threat are detected more readily than other facial expressions [74]. This early ERP difference between processing anger and pain facial expressions is unlikely to be the result of differences in low-level motion properties between the two expressions, as motion onset and overall motion did not differ across expressions (see Method). Moreover, the early ERP differences between the facial expressions were not evident in the infant group even though they watched the same stimuli and at this age possess similar visual acuity to adults [75]. The absence of an EPN in 8-month-old infants is in line with prior work showing that it is not until 12 months of age that infants show an enhanced EPN over posterior (occipital) electrodes in response to angry faces [33].

Our analysis further revealed significant amplitude differences between processing dynamic expressions of pain and anger for the LPP at frontal and central electrodes in adults. Specifically, the LPP was enhanced in its amplitude in response to pain expressions when compared to anger expressions. This replicates prior adult ERP findings using static expressions of pain [17] and suggests that adults show an increased evaluation and emotional arousal in response to seeing others in pain [22]. Supporting the notion that painful expressions evoked greater arousal in adults, subjective behavioral ratings of facial expressions revealed that pain faces are judged as being more arousing than angry faces. Taken together, these findings in adults are in agreement with other studies showing higher arousal ratings for pain faces compared to other negative expressions (e.g., anger and fear) [15], [17], [18] and also correspond to ERP work indicating arousal modulation effects on the LPP with more arousing stimuli eliciting greater LPPs [19].

Infants watching the same expressions showed ERP responses at frontal and central electrodes that were different from what was observed in adults. Specifically, angry expressions when compared to pain expressions elicited an enhanced negative component at frontal and central electrodes between 500 and 600 ms in 8-month-old infants. This finding is consistent with previous work in which 7-month-old infants showed a similar fronto-central ERP enhancement in response to static angry faces when compared to fearful faces [35]. Thus, these ERP data demonstrate that infants at 8 months of age are able to discriminate between facial expressions of pain and anger, and seeing angry expressions results in a greater allocation of attentional resources than seeing expressions of pain. Such an early developing attentional sensitivity as indexed by the increased ERP sensitivity to anger might be particularly critical when it comes to detecting potential sources of aggression and threat [26]–[28]. However, prior work suggests that only at around one year of age do infants begin to show adult-like neural processes that indicate threat detection from angry faces as reflected in the EPN [33]. Moreover, the observed differences in the infant and adult ERP responses to pain suggest that there is developmental change that occurs sometime after 8 months that sensitizes children to facial expressions of pain. This is in line with a host of behavioral work showing that empathic responding by helping and comforting others in pain only emerges later during ontogeny, namely, during the second year of life [40], [41]. It might therefore be particularly important in the future to extend the current paradigm by testing infants in their second year of life.

Our analysis further revealed that the amplitude of the ERP responses to angry facial expressions at frontal and central electrodes in adults was negatively correlated with their self-reported perspective-taking score on the IRI. Specifically, the higher adults rated themselves as possessing the ability and motivation to relate to others and to understand others' perspectives, the smaller the LPP amplitude to angry expressions.

In infants the ERP response to angry expressions was negatively correlated with their recovery from distress on the IBQ-R. Specifically, the better the infants were able to self-regulate their emotions (distress) as judged by their parents, the greater the negative amplitude of the ERP response to angry expressions. Although we expected IBQ-R subscales representing negative emotionality to be related to infants' ERP responses to negative facial expressions, no associations between these measures were found. Our results are similar to the findings by Martinos and colleagues [57] showing that only infants' self-regulation abilities but not infants' negative emotionality per se was associated with infants' ERP responses to negative (fearful) emotional expressions. In line with our findings, Martinos and colleagues [57] showed that infants that were better at self-regulation showed a larger Nc response to fearful facial expressions.

The finding that behavioral ratings of perspective-taking in adults and emotional self-regulation in infants correlated with the ERP response to angry faces suggests that sensitive responding to angry faces as a signal of interpersonal threat may afford specific self-regulatory mechanisms and that this ability to self-regulate/take the perspective of others may vary systematically across individuals. Moreover, it may further indicate that, in line with prior work [76]–[78], there is a developmental link between emotion regulation in infancy and perspective-taking later in life. However, this possible link should be assessed more explicitly in future work employing a longitudinal design.

These findings are in line with theoretical proposals in the empathy literature that have postulated a link between emotion (self) regulation and perspective-taking [78], [79] and with empirical findings with adults that show the influence of perspective-taking on anger regulation [80]. However, while these data may provide preliminary correlational evidence for this potential link, longitudinal work would be required to assess this association and its developmental trajectory systematically.

Infants and adults showed opposite patterns in their frontal EEG alpha asymmetry responses to pain and anger, suggesting developmental changes in the motivational processes engendered by the perception of these expressions. While pain resulted in greater relative left frontal activation in infants, indexing a motivational tendency to approach, adults showed a greater relative right frontal activation, indexing a motivational tendency to withdraw. Critically, in adults, greater relative right frontal activation to pain was correlated with a higher score of empathic concern. This might indicate a higher degree to which adults participate in other people's emotions and experience feelings of sympathy or concern for others in pain or distress [63], [64]. In other words, adults who judged themselves as having higher dispositional empathic concern responded with greater withdrawal to expressions of pain, indicative of a vicarious experience of the aversiveness of another person's pain [40], [81]. This effect observed in adults is in line with a host of studies emphasizing the role of experiencing another person's pain in empathic understanding [6], [82]. Another explanation for the observed greater relative right frontal activation during the observation of pain in the adult group is that, as suggested by prior work [23]–[25], painful expressions might be perceived as threatening. Therefore, the resulting motivational tendency in adults might have been to withdraw from the painful expression.

In contrast to adults, 8-month-old infants showed greater relative left frontal activation, indicating a tendency to approach expressions of pain. This suggests that infants do not experience another person's pain expression as aversive or negative but might rather be interested in the expression possibly to gather more information concerning the person's situation. While prior work measuring infants' behavioral responses to distress vocalizations and distress/pain simulations demonstrates that feelings of empathic concern already emerge in the first year of life [83], [84], our data suggest that infants at 8 months of age do not yet respond empathically to facial expressions of pain. This might have something to do with differences between vocally and facially expressed emotions and signs of vocal distress, as witnessed by infants in prior studies [83], [84], being a more direct and more powerful trigger of early forms of emotional and empathic responding [85]. Nevertheless, the ability to differentiate facial expressions of pain from other emotional facial expressions forms a prerequisite for the further development of empathy-related responding.

For an infant to experience an approach tendency towards facial expressions of pain as suggested by the current frontal EEG alpha asymmetry findings might provide an important mechanism to gather further information concerning the person's situation and may hence serve an important learning function.

The perception of angry faces resulted in greater relative left frontal activation in adults, indexing a motivational tendency to approach, while infants showed a greater relative right frontal activation to angry faces indexing a motivational tendency to withdraw. Our finding of greater relative left frontal activation in adults during the perception of angry faces is in line with prior work that obtained similar EEG asymmetry patterns when adults were experiencing anger themselves [54], [86], suggesting that perceiving and expressing anger may result in approach behaviors. The opposite pattern, relatively greater right frontal activation during the perception of angry faces, was found in the infant group, pointing to a developmental difference in the motivational evaluation of angry faces between infants and adults. This developmental difference may be explained by the fact that adults might respond to seeing angry faces as conveying interpersonal threat that elicits aggressive (attack) tendencies resulting in approach tendencies, whereas infants might feel frightened by an adult looking at them angrily resulting in withdrawal tendencies.

The developmental differences between infants and adults evident in the frontal EEG alpha asymmetry findings are consistent with the current ERP findings that indicate similar differences across ages, pointing to a general developmental change in responding to emotional expressions of pain and anger. More specifically, our data suggest that between infancy and adulthood there is considerable change when it comes to (a) discriminating between pain and anger as evident in the ERP responses and (b) perceiving the significance of these emotions for motivational brain systems as evident in the frontal EEG alpha asymmetry. This suggests that only through extensive experience with these facial expressions and the associated situations can a deeper understanding of these emotions be achieved [87]. As alluded to above, when exactly this development is achieved remains an open question and should be addressed in future studies with older infants or toddlers.

With regard to the expected correlation between infants' temperament measures and frontal EEG alpha asymmetry responses, no such correlations were found in the present study. This appears to be in contrast to a previous study by LoBue et al. [58] that reported correlations between approach and withdrawal-related temperament traits and EEG alpha asymmetry in 7- to 9-month-old infants. However, LoBue and colleagues [58] only found such correlations when they looked at frontal EEG asymmetry collapsed across emotion and neutral conditions but not when examining correlations for the experimental conditions separately, which were negative (e.g., threatening stimuli, such as snakes) and positive (e.g., non-threatening stimuli, such as giraffe) visual stimuli. Taken together, our findings and the findings from LoBue and colleagues [58] therefore suggest that there are no emotion-specific associations between frontal EEG asymmetry patterns and infants' temperament.

There are a few limitations of this study that require discussion. First, it should be noted that in the current study behavior was not measured directly to assess approach and withdrawal tendencies in infants and adults. Therefore, the present findings are limited to neural indexes of motivational tendencies and future research is needed to examine to what extent the brain measures correlate with overt behavioral responses. Second, with respect to the correlational analysis it should be acknowledged that the sample size is relatively small for a study investigating individual differences in emotion processing and that the measures used rely on self-report in the case of the adults and parental report in the case of the infants, which are prone to reporting biases. It would thus be important to further investigate the obtained individual differences by including more direct measures of temperament and empathy and correlate them with emotion processing in a larger sample across development.

In summary, it can be concluded that exploring the neural processes that underpin infants' and adults' responses to pain and anger has provided important insights into the nature of emotion perception and particularly its developmental and individual differences. Our data suggest that processing expressions of pain and anger is shaped by developmental changes that occur in the context of individual differences in emotional sensitivity that can be detected already very early on in ontogeny. Furthermore, the current study demonstrates that it is critical to utilize novel methodological approaches using multiple methods and measures in order for developmental differences to be uncovered and better understood.

## Supporting Information

Figure S1 **Adult event-related brain potentials.** This figure shows the event-related potentials of adults in response to facial expressions.
(TIF)

Figure S2 **Infant event-related brain potentials.** This figure shows the event-related potentials of infants in response to facial expressions.
(TIF)

Table S1 **Means of adult amplitudes.** Means of adult amplitudes in response to facial expressions in the time range of 250 to 350 ms at occipital electrodes (O1, O2).
(TIF)

Table S2 **Means of adult amplitudes.** Means of adult amplitudes in response to facial expressions in the time range of 400 to 500 ms at fronto-central electrodes (F3, Fz, F4, C3, Cz, C4).
(TIF)

Table S3 **Means of infant amplitudes.** Means of infant amplitudes in response to facial expressions in the time range of 200 to 300 ms at occipital electrodes (O1, O2).
(TIF)

Table S4 **Means of adult amplitudes.** Means of infant amplitudes in response to facial expressions in the time range of 500 to 600 ms at fronto-central electrodes (F3, Fz, F4, C3, Cz, C4).
(TIF)

Table S5 **Mean adult lateralization scores (log-transformed) in response to facial expressions.**
(TIF)

Table S6 **Mean infant lateralization scores (log-transformed) in response to facial expressions.**
(TIF)
