# Supplementary figures and images for: Developmental and Individual Differences in the Neural Processing of Dynamic Expressions of Pain and Anger
Source: PLoS One. 2014 Apr 4;9(4):e93728. doi: 10.1371/journal.pone.0093728 (PMC3976316; doi:10.1371/journal.pone.0093728)

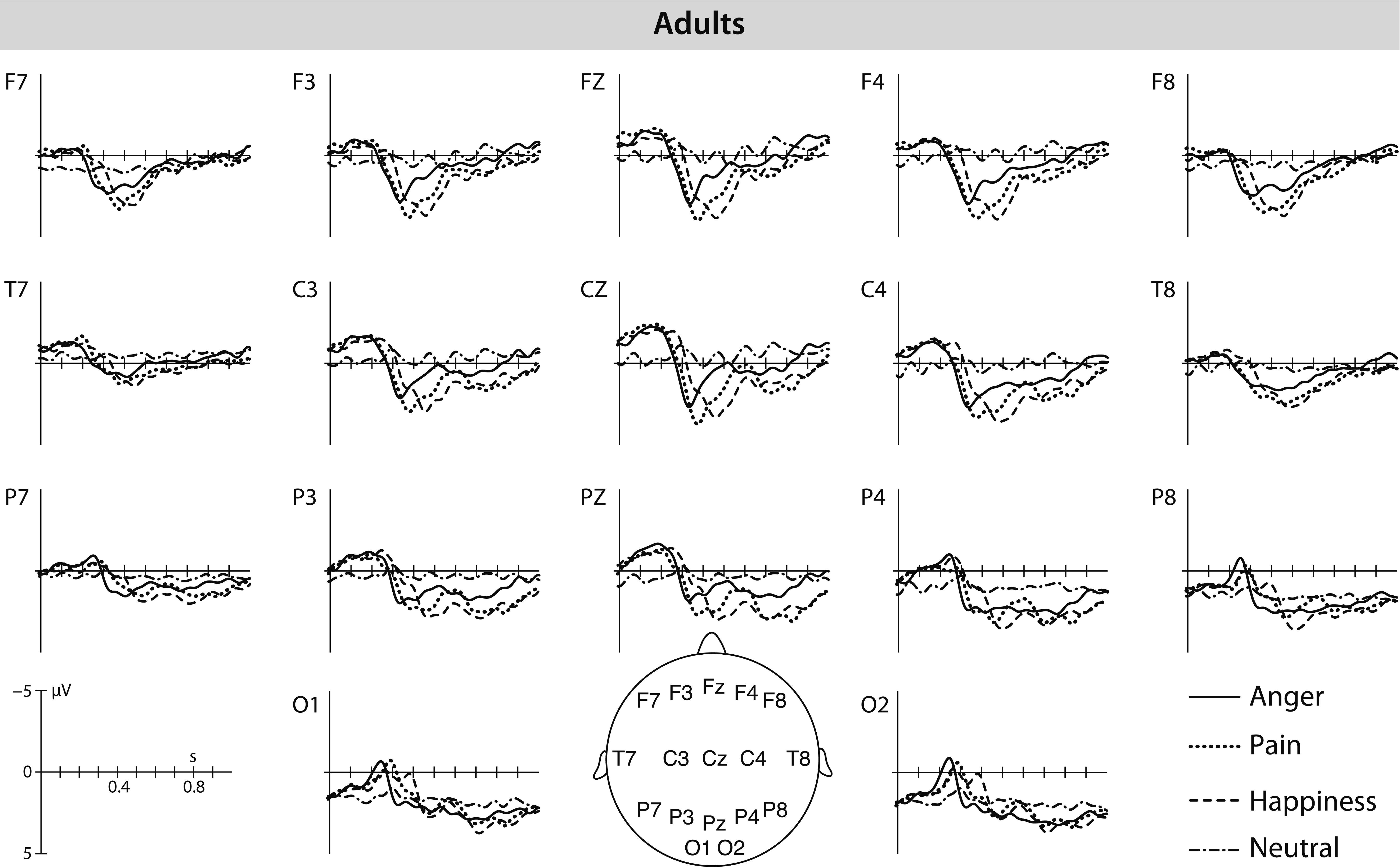

Supplement: Figure S1 — Adult event-related brain potentials. This figure shows the event-related potentials of adults in response to facial expressions. (TIF) [file pone.0093728.s001.tif]

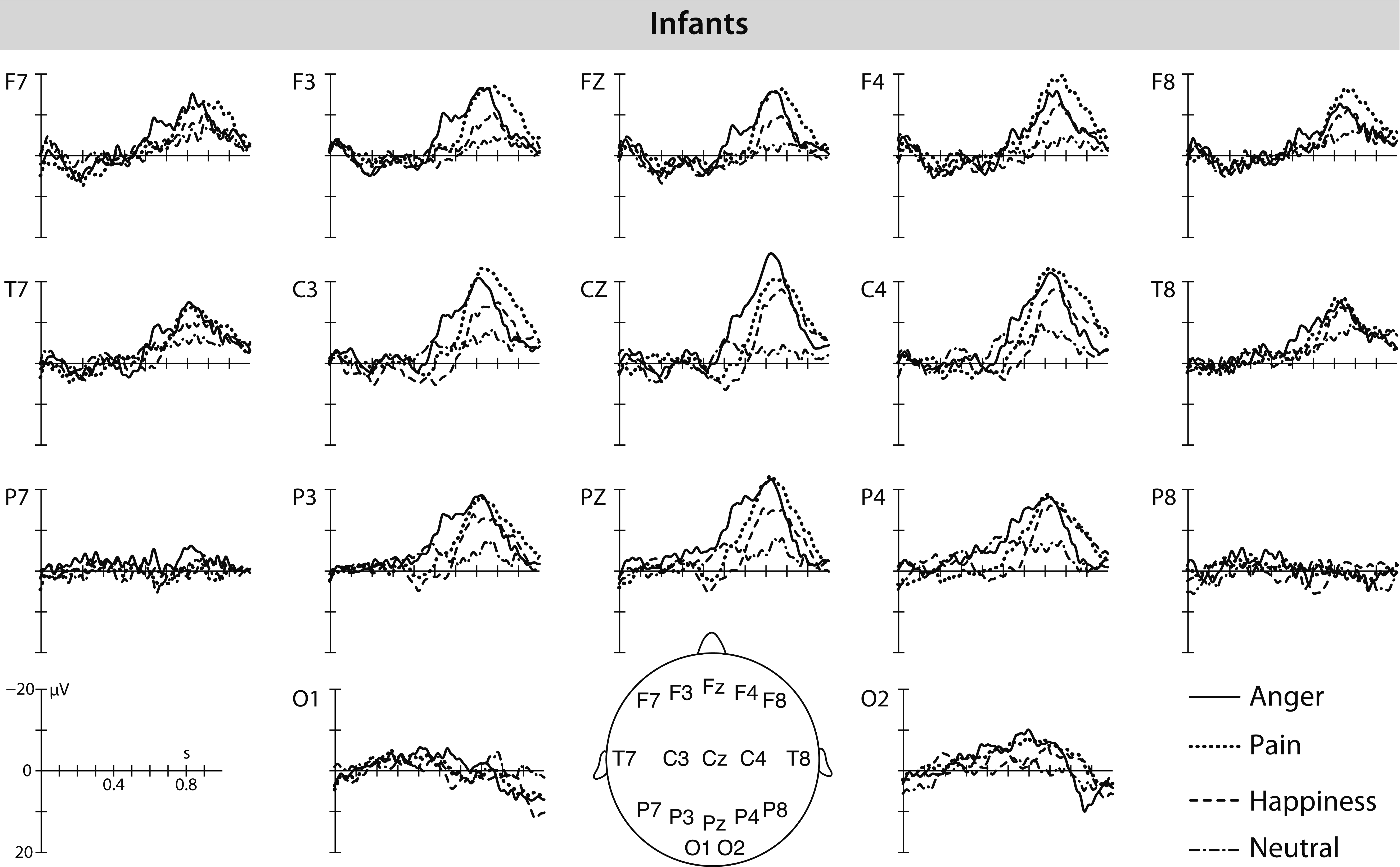

Supplement: Figure S2 — Infant event-related brain potentials. This figure shows the event-related potentials of infants in response to facial expressions. (TIF) [file pone.0093728.s002.tif]

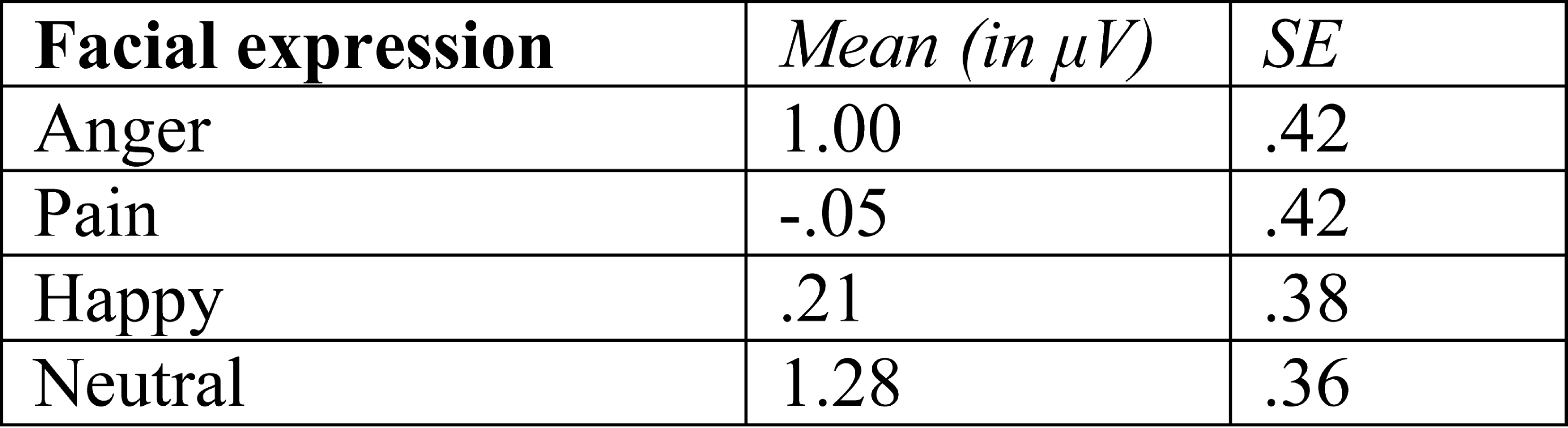

Supplement: Table S1 — Means of adult amplitudes. Means of adult amplitudes in response to facial expressions in the time range of 250 to 350 ms at occipital electrodes (O1, O2). (TIF) [file pone.0093728.s003.tif]

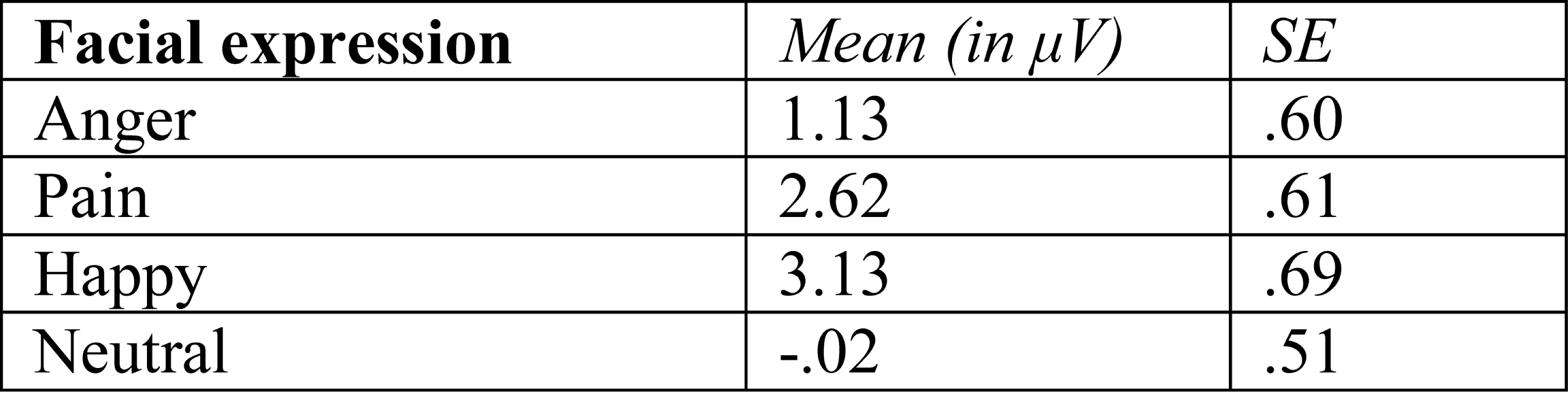

Supplement: Table S2 — Means of adult amplitudes. Means of adult amplitudes in response to facial expressions in the time range of 400 to 500 ms at fronto-central electrodes (F3, Fz, F4, C3, Cz, C4). (TIF) [file pone.0093728.s004.tif]

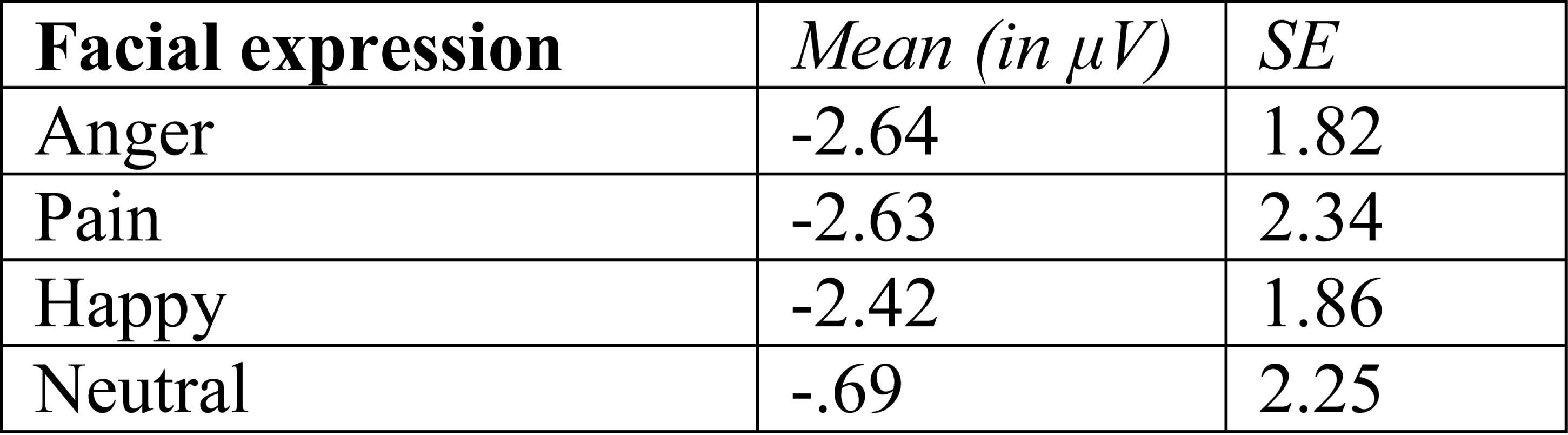

Supplement: Table S3 — Means of infant amplitudes. Means of infant amplitudes in response to facial expressions in the time range of 200 to 300 ms at occipital electrodes (O1, O2). (TIF) [file pone.0093728.s005.tif]

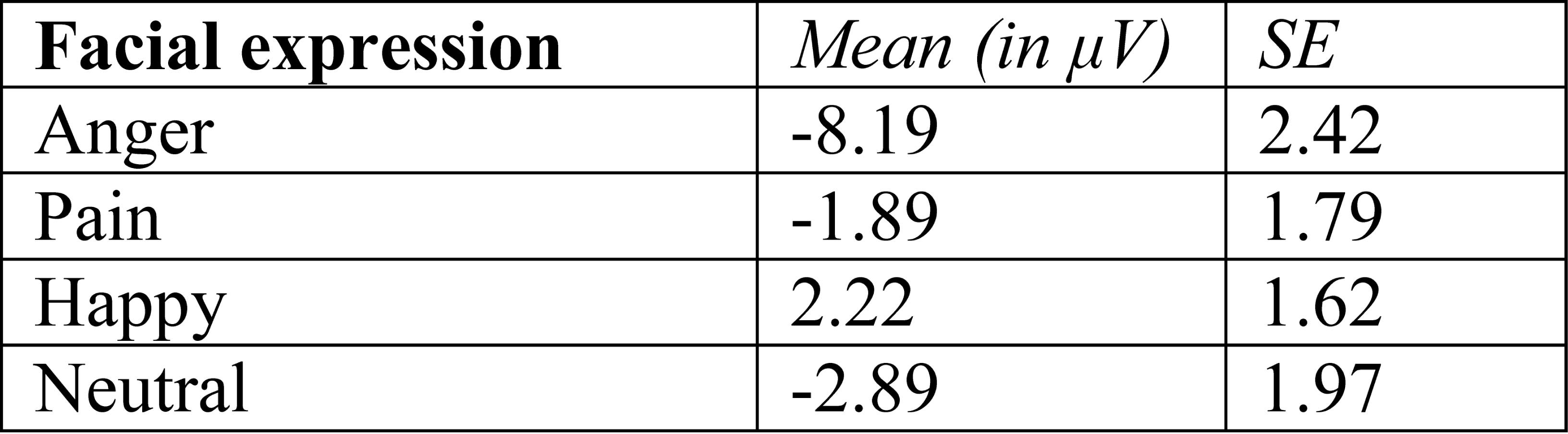

Supplement: Table S4 — Means of adult amplitudes. Means of infant amplitudes in response to facial expressions in the time range of 500 to 600 ms at fronto-central electrodes (F3, Fz, F4, C3, Cz, C4). (TIF) [file pone.0093728.s006.tif]

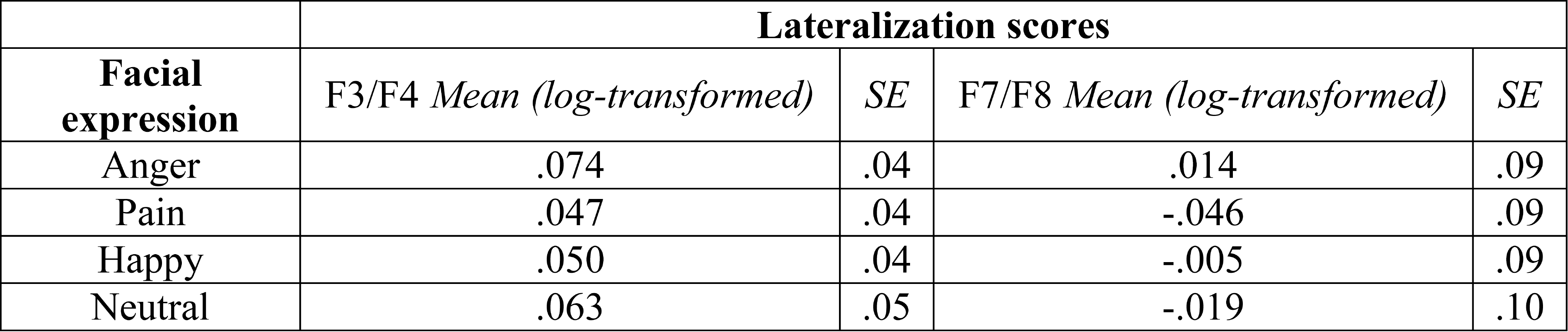

Supplement: Table S5 — Mean adult lateralization scores (log-transformed) in response to facial expressions. (TIF) [file pone.0093728.s007.tif]

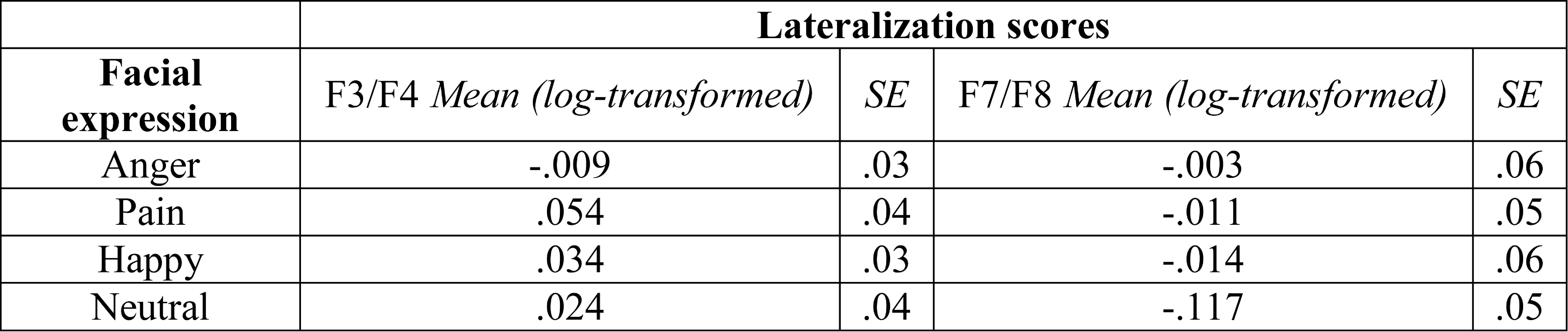

Supplement: Table S6 — Mean infant lateralization scores (log-transformed) in response to facial expressions. (TIF) [file pone.0093728.s008.tif]
